# Transcription tipping points for T follicular helper cell and T-helper 1 cell fate commitment

**DOI:** 10.1038/s41423-020-00554-y

**Published:** 2020-09-30

**Authors:** Amania A. Sheikh, Joanna R. Groom

**Affiliations:** 1 Divisions of Immunology and Molecular Immunology, Walter and Eliza Hall Institute of Medical Research, Parkville, VIC 3052 Australia; 2 Department of Medical Biology, University of Melbourne, Parkville, VIC 3010 Australia

**Keywords:** Cytokines, Infection, T follicular helper cells, T-helper 1 cells, Transcription factors, Lymphocyte differentiation, Viral infection

## Abstract

During viral infection, immune cells coordinate the induction of inflammatory responses that clear infection and humoral responses that promote protection. CD4^+^ T-cell differentiation sits at the center of this axis. Differentiation toward T-helper 1 (Th1) cells mediates inflammation and pathogen clearance, while T follicular helper (Tfh) cells facilitate germinal center (GC) reactions for the generation of high-affinity antibodies and immune memory. While Th1 and Tfh differentiation occurs in parallel, these CD4^+^ T-cell identities are mutually exclusive, and progression toward these ends is determined via the upregulation of T-bet and Bcl6, respectively. These lineage-defining transcription factors act in concert with multiple networks of transcriptional regulators that tip the T-bet and Bcl6 axis in CD4^+^ T-cell progenitors to either a Th1 or Tfh fate. It is now clear that these transcriptional networks are guided by cytokine cues that are not only varied between distinct viral infections but also dynamically altered throughout the duration of infection. Thus, multiple intrinsic and extrinsic factors combine to specify the fate, plasticity, and function of Th1 and Tfh cells during infection. Here, we review the current information on the mode of action of the lineage-defining transcription factors Bcl6 and T-bet and how they act individually and in complex to govern CD4^+^ T-cell ontogeny. Furthermore, we outline the multifaceted transcriptional regulatory networks that act upstream and downstream of Bcl6 and T-bet to tip the differentiation equilibrium toward either a Tfh or Th1 fate and how these are impacted by dynamic inflammatory cues.

## Introduction

CD4^+^ T cells form a bridge between the cell-mediated and humoral arms of the adaptive immune response to pathogens. Following infection, naive CD4^+^ T cells can differentiate into distinct T-helper (Th) subsets, including Th1, T follicular helper (Tfh), Th2, Th17, and regulatory T cells as well as memory cell precursors.^1^ Naive and newly activated CD4^+^ T cells sense changes in the microenvironment and integrate those signals through the upregulation of lineage-defining transcriptional factors. The balance of these transcription factors then directs CD4^+^ T cells down a particular development path. The flexibility of CD4^+^ T cells to diverge into distinct subsets, which is guided by pathogen-specific inflammatory cues, enables tailored immune responses against diverse immune challenges.

Tfh and Th1 cells are key players in orchestrating CD4^+^ T-cell-dependent cell-mediated and humoral adaptive immune responses to intracellular pathogens, such as viruses. Tfh cells migrate into germinal centers (GCs), which are specialized microanatomical structures that form in secondary lymphoid organs following vaccination and infection and are sites of robust humoral immune responses. Within GCs, Tfh and B cells interact to allow affinity maturation of B cells and their differentiation into memory and high-affinity antibody-secreting B cells. Dysregulated Tfh cell differentiation profoundly impacts immune responses and can lead to immunodeficiency and systemic autoimmune disease.^2^

In contrast, cell-mediated immune responses during viral infection are elicited by Th1 cells. Unlike Tfh cells, Th1 cells leave secondary lymphoid organs to infiltrate peripheral tissues and facilitate cell-mediated responses to localized inflammation or infection. Here, they produce cytokines, which in turn activate macrophages and CD8^+^ T cells to promote clearance of intracellular pathogens and tumors.^3^ Given this role in orchestrating the humoral and cellular arms of the adaptive response, a fundamental goal is to understand the factors that initiate Tfh and Th1 ontogeny.

**Fig. 1:**
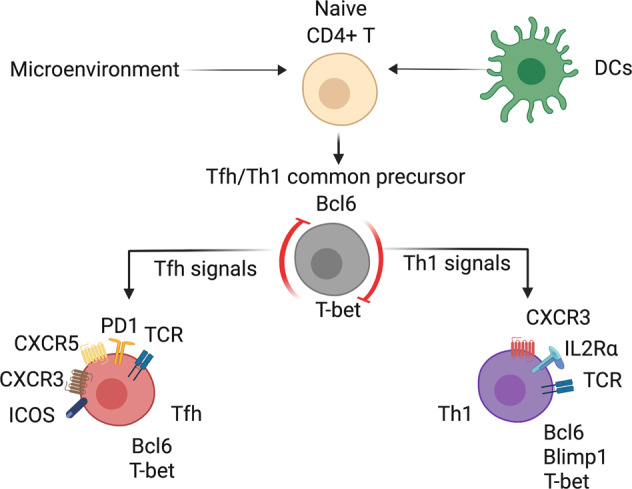
Tfh and Th1 fate trajectories. Naive CD4^+^ T cells following antigen presentation by dendritic cells and additional signals from the microenvironment develop into common Tfh/Th1 precursor cells that coexpress Bcl6 and T-bet, which are expressed competitively. In the presence of Tfh signals, common Tfh/Th1 precursor cells differentiate into Tfh cells that co-express key transcription factors (Bcl6 and T-bet), chemokine receptors (CXCR5 and CXCR3), and molecules (PD-1 and ICOS). In contrast, common Tfh/Th1 precursor cells that differentiate into Th1 cells in response to Th1 signals express the canonical Th1 transcription factors Blimp-1, T-bet, and Bcl6; chemokine receptors (CXCR3); and IL-2 receptor (IL-2Rα)

In Th1-skewed infections, CD4^+^ T cells differentiate into Tfh cells, Th1 cells, and memory cell populations in parallel. The choice between these fates follows antigen presentation by DCs and is based on T-cell receptor signal strength, the microenvironment and costimulatory receptor signaling induced by cellular interactions. Prior to this developmental bifurcation and the formation of mature effector subsets, Tfh and Th1 cells share a common precursor stage (Fig. 1);^4,5^ therefore, the differentiation path of Tfh and Th1 cells overlaps, and CD4^+^ T cells select either fate at the expense of the other. Currently, the precise timing of this CD4^+^ T-cell bifurcation is contested. While some studies indicate that CD4^+^ fate decisions are imprinted early, prior to the first cell division following activation,^6,7^ others indicate that this branching occurs later, between days 2 and 4 post infection.^5^ These discrepancies may represent differences in infection models, experimental approaches to define precursors, or plasticity in differentiating CD4^+^ T cells. In this review, we discuss how transcriptional factors work independently and together to direct Tfh/Th1 bifurcation. We propose that a Bcl6–T-bet axis exists in parallel with the Bcl6–Blimp-1 paradigm for Tfh/Th1 ontogeny. Importantly, these transcriptional networks are context-dependent and tuned by dynamic changes in the environment to tip the balance toward either Tfh or Th1 cell formation. Furthermore, we discuss recent advances and anomalies in the field that shed new light on how the unique cytokine milieu in different infections is a key decisive factor in determining Tfh versus Th1 fate. Understanding the multifactorial process of the Tfh/Th1 dichotomy will pave the way to rationally develop immunotherapeutics to direct pathogen clearance and vaccines that promote the formation of neutralizing antibodies following viral infections.

## The Bcl6–T-bet axis: the linchpin of Tfh/Th1 equilibrium

Despite being phenotypically and functionally distinct, Tfh and Th1 cells share precursors characterized by the coexpression of two lineage-specifying transcriptional factors, Bcl6 and T-bet (Fig. 1).^4,5,8–11^ Unlike mature effector populations, CD4^+^ T-cell memory precursors express Bcl6 and T-bet at low to intermediate levels, and Bcl6 deficiency leads to loss of CD4^+^ central memory cells along with Tfh cells.^12–15^ Within the precursors of effector cells, Bcl6 and T-bet are competitively co-expressed and antagonize the expression of each other. Ultimately, one of these transcription factors wins the differentiation race to determine the formation of Tfh or Th1 cells. It is therefore important to understand the characteristics that allow these transcriptional factors to either be co-expressed or show dominate expression to mediate CD4^+^ T-cell fate commitment in the context of infection.

### Bcl6

Bcl6 is a proto-oncogene zinc-finger transcriptional repressor that has a profound role in the function and differentiation of multiple immune lineages. Bcl6 expression is essential for robust humoral immunity. GC B cells express high levels of Bcl6, which prevents their differentiation into plasma and memory cells.^16,17^ Consistent with this, Bcl6-deficient mice display impaired GC formation, lack of antigen-specific antibodies against T-cell-dependent antigens, and impaired affinity maturation.^18^ In addition, Bcl6 regulates the generation and maintenance of memory CD8^+^ T cells.^19^ A decade ago, studies illustrated the role of Bcl6 in Tfh fate commitment.^2^ Initial studies highlighted that the interleukin 6 (IL-6) and interleukin 21 (IL-21) cytokines promote Tfh differentiation in vitro and that this differentiation is associated with the upregulation of Bcl6 in both murine and human Tfh cells.^11,20–22^ CD4^+^ T cells deficient in Bcl6 failed to develop into Tfh cells and were insufficient to support GC reactions in vivo, thus definitively showing that Bcl6 is required for Tfh differentiation.^11,21,23^ Since the discovery of Bcl6 as a lineage-defining transcription factor, studies on the Tfh transcriptional differentiation axis have been focused on the antagonistic relationship between Bcl6 and Blimp-1, (a transcription factor encoded by *Prdm1*).^11,21,23,24^ Bcl6 and Blimp-1 antagonize and inversely regulate each other’s expression in both GC B cells and Tfh cells. In T cells, Blimp-1 is downregulated in Tfh cells but it is maintained at high levels in non-Tfh CD4^+^ T cells.^11^ The overexpression of Blimp-1 inhibits CD4^+^ T cells to acquire the Tfh phenotype by inhibiting the expression of canonical markers, including CXCR5, ICOS, and PD-1.^11,21,23,25^ While it was previously proposed that Tfh cell formation was a default differentiation state for CD4^+^ T cells, this has recently been shown to not be the case, as cells deficient in both Bcl6 and Blimp-1 fail to form Tfh cells in vivo following both immunization and viral infection.^25^ As discussed below, this study confirmed previous work highlighting that Bcl6 acts as a hub for the transcriptional repression of pathways that inhibit Tfh differentiation.^25^ Importantly, this and other studies have recently demonstrated that Bcl6 repression of these transcriptional networks occurs independently of Blimp-1, further highlighting the indispensable role for Bcl6 in Tfh fate commitment.^25,26^

Several unique structural features of the Bcl6 protein allow it to interact with diverse transcription factors and chromatin modifiers to form transcriptional complexes. These interactions permit Bcl6 to control gene expression in CD4^+^ T-cell precursors and in mature Tfh cells. The Bcl6 protein consists of an N-terminal POZ (or BTB, broad complex, tramtrack, bric-a-brac) domain, a middle domain also known as RDII, and a C-terminal zinc-finger domain. The BTB domain mediates interactions with Bcl6 corepressors, including N-COR, SMRT, and BCOR.^27–29^ These cofactors compete to bind to the Bcl6 N-terminus and can recruit histone deacetylase (HDAC) protein complexes to form a transcription-repressing complex at the target gene. HDACs are enzymes that modify chromatin structure, and this in turn prevents the ability of transcription factors to bind to regulatory regions and activate the transcription of target genes.^30–32^ Mutations in the BTB domain inhibit the differentiation of Tfh cells.^33^ Furthermore, BCOR-deficient CD4^+^ T cells fail to differentiate into Tfh cells.^34^ Taken together, these results suggest that mutations in the BTB domain prevent BCOR binding. While N-COR and SMRT are also expressed in CD4^+^ T cells, further research is needed to determine their role in Tfh differentiation; however, recently, N-COR was shown to be recruited to the Bcl6 promoter to negatively regulate Bcl6 expression.^25^ Bcl6 utilizes its largest domain, the RDII domain, to associate with the corepressor MTA3. This interaction leads to the repression of Blimp-1.^35^ The C-terminus of the Bcl6 protein harbors six Kruppel-like C2H2-type zinc-fingers that can bind to a nine-base-pair DNA sequence (TTCCT(A/C)GAA) that shares sequence homology with STAT (signal transducer and activator of transcription) and activator protein 1 (AP-1) DNA binding sites.

In 2015, a landmark study led by Hatzi et al. mapped the cis-acting targets of BCL6 in human GC Tfh cells. This research outlined that BCL6 is directly or indirectly recruited to loci of multiple key genes actively involved in Tfh fate.^22^ First, BCL6 is indirectly recruited to non-BCL6 DNA binding sites by other transcription factors in Tfh cells.^22^ In accordance, BCL6 is enriched at AP-1 DNA binding motifs in Tfh cells. This is mediated by the physical association of BCL6 and AP-1 in CD4^+^ T cells, which enables BCL6 to be recruited to cis-regulatory regions of many genes in an AP-1-dependent manner.^22^ AP-1 is a collective term used for transcription factors that consist of Jun, Fos, or ATF (activating transcription factor) subunits that form dimers. These transcriptional activators play a vital role in T effector cell differentiation, proliferation, and function.^36–38^ It is suggested that recruitment of BCL6 to the AP-1 DNA binding motif converts AP-1-dependent gene activation to repression. Notably, BCL6 and AP-1 colocalize at the *Prdm1* locus, which contains an AP-1 DNA binding motif.^22^ It could be that BCL6 exploits AP-1 to establish the Tfh transcription program through suppression of Blimp-1. A secondary mechanism of BCL6 occurs via direct binding to the enhancer and promoter regions of genes important in T-cell migration. The relocation of Tfh precursor cells to the B-cell follicle is a prerequisite for an effective GC response.^39^ BCL6 regulates multiple T-cell migration factors to establish Tfh cell homing to B-cell follicles and to prevent Tfh cell egress from secondary lymphoid tissues. Specifically, BCL6 binds to the promoter and enhancer of *Ccr7* (encoding CCR7) and *Selplg* (encoding PSGL-1 proteins), which are known to regulate the migration of T cells to the T zone of secondary lymphoid tissues.^22^ *Selplg* was shown to be directly repressed by Bcl6 following LCMV infection.^25^ Furthermore, BCL6 binds to the gene encoding EBI2, which may lead to repression of its expression.^22^ In both B and Tfh cells, EBI2 has been shown to play a role in the localization of cells to the extrafollicular regions of secondary lymphoid tissues.^40,41^ Moreover, BCL6 promotes the expression of the key Tfh cell markers IL-21R and CXCR5 in CD4^+^ T-cell culture, and mutations in the Bcl6 zinc-finger DNA binding domain restrict BCL6-mediated upregulation of Bcl6, IL-21R, and CXCR5 in CD4^+^ T cells.^21^ Recently, Bcl6 repression of Gata3, Runx2, and Klf2 was confirmed to increase the expression of CXCR5 to promote the migration of CD4^+^ cells into B-cell follicles in vivo.^25^ Overall, these actions of BCL6 on T-cell migration facilitate the movement of cells toward the follicle, into environmental niches that further promote Tfh differentiation.

One of the most critical roles of Bcl6 in imprinting Tfh fate is to block the differentiation of alternate Th cell types. For example, in human Tfh cells, BCL6 binds to the promoter regions of genes important for alternate Th fates, including *GATA3*, *RORA*, and *IFNGR1*, and the enhancer regions of the *TBX21* gene (which encodes T-bet).^22^ In addition, Gata3, Tbx21, and Id2 constitute a transcriptional signature of Bcl6-repressed genes in antigen-specific mouse T cells.^25^ Mature CD4^+^ T cells also have BCL6 binding sites that are depleted of the enhancer histone marks H3K4me1 and H3K27ac in comparison to naive CD4^+^ T cells, suggesting that these regulatory regions are in an inactive state. It is likely that BCL6, along with its corepressors N-COR, SMRT, and BCOR, recruit HDACs to these sites to dynamically modify histone marks.^42^ Furthermore, Bcl6-deficient cells cultured in Th1 conditions demonstrated increased expression of T-bet and RORγt,^21^ suggesting that this mechanism may be at play even in non-Tfh cells. In summary, Bcl6 controls Tfh fate commitment via direct repression of alternative fates by regulating the coercion of cofactors and epigenetic factors and inhibiting alternate Th cell positioning and cytokine signaling. Together, these studies show that Bcl6 is highly involved in establishing Tfh fate.

### T-bet

The transcription factor T-bet is expressed in numerous immune lineages and plays an essential role in regulating antiviral immunity. In CD8^+^ T cells, T-bet preferentially promotes effector T-cell differentiation over memory precursor differentiation.^43^ B cells deficient in T-bet failed to produce IgG2a following acute and chronic viral infections.^44–46^ Furthermore, T-bet expression in GC B cells plays a role in the localization of these cells to the GC dark zone during malaria.^47,48^ In addition, T-bet is required for the differentiation of several ILC populations, including NK cells,^49–51^ in which it instructs interferon (IFN)-γ production.^52–54^ Despite these pleotropic roles, T-bet is best known for its essential function in Th1 cell differentiation and driving the production of the canonical Th1 cytokine IFN-γ.^55^ T-bet binding sites exist in the *Ifnγ* locus along with permissive H3K4me3 and H3K36me3 histone modifications in Th1 cells.^4^ T-bet also prevents Th cell precursors from adopting non-Th1 effector fates.^56,57^ Indeed, retroviral gene transduction of T-bet into Th2 cells converted cells to IFN-γ-producing Th1 cells,^55^ demonstrating that T-bet can direct Th1 fate in fully polarized non-Th1 helper cells. In addition, T-bet directly binds to the loci of the gene encoding tumor necrosis factor (TNF). TNF and IFN-γ are key cytokines regulating Th1 cell effector function. T-bet also regulates the expression of the chemokines CCL3 and CCL4 and the chemokine receptor CXCR3, which are indispensable for Th1 development and migration of Th1 cells to the site of inflammation.^3,58^ In addition to directly binding to these loci and activating their transcription, T-bet has been shown to bind hundreds of immune regulatory genes across the mouse and human genomes.^4,59–61^ Like all other T-box proteins, T-bet contains two functional domains.^62^ The T‐box domain, which binds a 24-bp palindromic DNA sequence, consists of the T-bet recognition sequence TCACACCT. The unique quaternary structure of T-bet enables the binding of two distinct DNA sites, potentially allowing T-bet to mediate DNA loop formation and long-range DNA interactions.^62^ Meanwhile, the transactivation domain facilitates the binding of T-bet-interacting proteins and transcriptional cofactors, such Mediator and P-TEFb, which are recruited to form the super elongation complex to activate Th1 gene expression.^61^ As described below, this domain also allows T-bet to coopt the function of other transcriptional regulators.

Understanding how T-bet imprints the Th1 gene program at the expense of an alternative Th cell program has been a topic of interest for more than a decade.^63^ T-bet encourages the Th1 fate by preventing alternate Th gene programs and negatively regulating the transcription of lineage-defining transcription factors and prototypical alternate Th genes. In addition to the previously mentioned co-expression of T-bet and Bcl6, T-bet can also be co-expressed with other lineage-specifying transcriptional factors in both precursor and committed cell subsets, such as its coexpression with RORγt in Th17 cells and with GATA3 in Th2 cells.^63^ In these settings, T-bet uses a similar mechanism to sequester these alternate Th cell transcriptional factors during in vitro T-cell differentiation.^56,57^ TCR signaling via tyrosine protein kinase (ITK) phosphorylates the motif at the C-terminal domain of T-bet. This promotes the formation of the T-bet-GATA3 complex in CD4^+^ T cells cultured in Th1-polarized conditions. As a result, GATA3 is sequestered in Th1 cells, which prevents GATA3 from activating the Th2 gene program.^56^ Furthermore, T-bet directly binds to the sites in the *Gata3* locus that are in close proximity to H3K27me3 repressive chromatin modifications, thus inhibiting Gata3 expression in Th1 cells.^59^ Unlike other lineage-defining transcription factors, GATA3 is expressed in naive CD4^+^ T cells,^64^ and its expression is substantially reduced in Th1 cells.^59^ In addition, T-bet-RUNX3 and T-bet-NFAT1 complexes limit RUNX3- and NFAT1-mediated Th2 signature cytokine expression (interleukin-2 (IL-2), IL-4, IL-5, and IL-13).^65,66^ Similarly, T-bet physically interacts with Runx1 in Th17-polarizing conditions. This blocks Runx1 binding to the *Rorc* promoter, thereby inhibiting its transcription. This in turn cripples RORγt-mediated Th17 differentiation.^57^ Thus, the constitutive expression of T-bet in Th cell precursors leads to reduced RORγt, blocking Th17 and promoting Th1 differentiation. T-bet in CD4^+^ T cells can directly bind to its own *Tbx21* locus, and this binding site is associated with permissive H3K4me1 histone modifications.^60^ However, T-bet induction is unchanged in T-bet-deficient cells when stimulated with IL-12 and IFN-γ and following *Toxoplasma gondii* infection.^59^ Conversely, the T-bet expression in T-bet and Stat4 double-deficient mice is substantially lower than that in Stat4-deficient mice during *Toxoplasma gondii* infection, suggesting that T-bet may regulate its own expression in certain circumstances.^59^

### Bcl6–T-bet complexes

Considering the multiple mechanisms that both Bcl6 and T-bet use to transcriptionally imprint CD4^+^ T cell differentiation, it may appear counterintuitive for these two factors to not only be competitively expressed but also work together to direct Tfh and Th1 differentiation and function. However, T-bet collaborates with Bcl6 to prolong non-Th1 helper cell program repression even in fully in vitro-differentiated Th1 cells.^8^ While it is possible that T-bet–Bcl6 complexes may bind to Bcl6 DNA binding sites, leading to the Tfh fate, it appears that T-bet dominates Bcl6 in these interactions and utilizes the transcriptionally repressive actions of Bcl6 to promote Th1 identity. This dominance occurs due to  the C-terminus of T-bet, which masks the Bcl6 DNA binding site while leaving the T-bet, T-box DNA binding domain exposed.^8^ Among others, the T-bet-Bcl6 complex can be recruited to the *Ifnγ* locus and *Socs1* and *Socs3* promoters in Th1 cells.^8^ As the name (suppressor of cytokine signaling) suggests, Socs1 is involved in blocking the IFN-γ and STAT1 signaling pathways. These signaling pathways are critical for acquisition of the Th1 gene program.^67,68^ However, following the establishment of the Th1 fate, T-bet-Bcl6 complexes act to decrease these signals. In this way, the recruitment of Bcl6 to the *Ifnγ* locus prevents an excessive amount of IFN-γ in Th1 culture.^8^ Potentially, this action may limit the immune pathology and autoimmunity caused by excessive Th1 signals; however, further studies are required to determine if this molecular mechanism is relevant in vivo and disrupted during immune pathology. In addition, Blimp-1 is highly expressed in Th1 cells, and as mentioned above, Blimp-1 antagonizes *Bcl6* expression. Therefore, Blimp-1 further prevents the expression of Bcl6 and Tfh genes in Th1 cells.^63^ Together, these findings unravel potential mechanisms implemented by T-bet to block non-Th1, and particularly Tfh, differentiation.

As discussed, Bcl6 is constitutively expressed in early Th1 precursors at low levels. Importantly, the reverse is also true, in that T-bet can be coexpressed with Bcl6 in precursor Tfh cells both in culture and during infection.^4^ Originally, it was suspected that T-bet coexpression was transient during Tfh differentiation; however, we and others have demonstrated high expression of T-bet in Tfh cells following infection.^5,46,69^ The molecular mechanisms of T-bet-Bcl6 coexpression are yet to be defined within Tfh cells. It is likely that they form functional complexes, similar to those described in Th1 cells, in these cells. Of note, Tfh cell expression of T-bet is required to regulate the expression of key Th1 factors, IFN-γ, and CXCR3, although a recent fate-mapping study demonstrated that Tfh cells could continue to express IFN-γ even when T-bet expression was transient.^46,69,70^ T-bet-deficient Tfh cells and their precursors promote B-cell isotype switching toward IgG1 during influenza infection, potentially through the loss of IFN-γ and other alterations of Tfh cytokine production.^46^

Intriguingly, we have demonstrated a context-specific role for T-bet in Tfh differentiation. Specifically, while distinct viral infections induce T-bet, the degree of this expression is distinct between infections. In settings in which low T-bet is induced, its loss results in the promotion of the Tfh lineage in vivo. In contrast, when T-bet is highly induced within Tfh cells, T-bet deficiency limits the formation of both Th1 and Tfh cells.^46^ This work highlights that the ratio of Bcl6 to T-bet within precursor cells is critical for precursor cell fate decisions. How these ratios are established by and interconnected with environmental signals and supporting transcriptional networks to instruct not only the fate commitment of CD4^+^ T cells but also their overall function, and impact on humoral immune responses remains to be determined.

## Transcriptional networks of the Tfh/Th1 dichotomy: fine-tuning the lineage-defining factors

### High mobility group (HMG) transcription factors

**Fig. 2:**
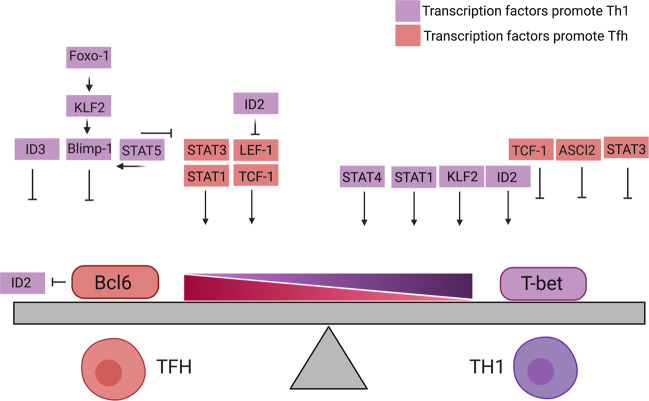
Transcriptional networks leading to Tfh and Th1 differentiation. The lineage-defining transcription factors Bcl6 and T-bet maintain equilibrium in Tfh/Th1 precursors. The interplay between a secondary set of transcription factors either promotes Bcl6 (*pink*) and T-bet (*purple*) or inhibits Bcl6 (*purple*) and T-bet (*pink*) expression, which tips the Bcl6–T-bet axis and directs Tfh and Th1 fate commitment

The interplay between multiple transcription factors is highly regulated and forms the backbone of T-cell differentiation. These multifaceted regulatory networks act both upstream and downstream of Bcl6 and T-bet to tip the differentiation equilibrium to bias either a Tfh or Th1 fate (Fig. 2). Key regulators of the Bcl6–T-bet axis are the HMG family of transcription factors, T-cell factor (TCF-1), and lymphoid enhancer factor (LEF-1). TCF-1 is encoded by the *Tcf7* gene, and LEF-1 is encoded by *Lef1*. Several studies delineate the profound role of TCF-1 and LEF-1 in T-cell responses. Both TCF-1 and LEF-1 support memory CD8^+^ T-cell formation by the expression of another T-box protein, Eomes.^71,72^ In addition, TCF-1 favors the expression of GATA3, a canonical Th2 transcription factor.^73^ It has also been demonstrated that TCF-1 dampens the inflammatory effects of Th17 cells by reducing IL-17A expression.^74^ This implies that HMG transcription factors favor a non-effector T-cell phenotype. In keeping with these observations, TCF-1 and LEF-1 expression promotes Tfh development. TCF-1 and LEF-1 are highly expressed in naive CD4^+^ T cells, and they remain high in Tfh precursors and mature Tfh cells during LCMV, vaccinia virus, and blood stage *Plasmodium* infection in mice.^5,75,76^ In contrast, TCF-1 and LEF-1 are rapidly downregulated in effector CD8^+^ and Th1 T cells.^75–77^ While culture of CD4^+^ T cells in either Tfh-polarizing (αIFN-γ, αIL-12, and rmIL-6) or Th1-polarizing (αIL-4, αTGF-β, and rmIL-12) conditions did not result in changes in Lef1 or Tcf7 transcription,^75^ in CD8^+^ T cells, the downregulation of TCF-1 in effector cells compared with memory precursors was driven by IL-12.^78^ This indicates that either there are differences in IL-12 signaling between CD4^+^ and CD8^+^ T cells or that IL-12 works with other factors to independently instruct between Th1/Tfh and effector/memory differentiation. Thus, additional studies are needed to resolve these differences.

TCF-1 instructs Tfh differentiation through multiple mechanisms. First, TCF-1 directly promotes *Bcl6* and inhibits *Prdm1* transcription in Tfh cells during LCMV infection.^76,79^ Second, TCF-1 is enriched at the IL-6 receptor gene locus (*Il6rα* and *Il6st*).^75^ This likely enables enhanced responsiveness of Tfh cells to IL-6 signaling, which is important in Tfh differentiation.^80^ In accordance, TCF-1-deficient mice demonstrated reduced expression of other factors that are involved in Tfh cell commitment and growth, including Bcl6 and ICOS. Conversely, loss of TCF-1 promoted the expression of Th1 cell-associated factors in Tfh cells, including T-bet, Blimp-1, and CD25 protein.^75,76^ Finally, dually expressed p33 (an isoform of TCF-1) and Bcl6 proteins physically interact and act to recover Bcl6-mediated autorepression.^76^ However, whether Bcl6 forms a heterodimer with p33 or Bcl6-p33 recruits another transcriptional regulator to reverse Bcl6 autoregulation in the context of viral infection remains unclear. Although some of the mechanisms by which TCF-1 promotes Tfh differentiation act side-by-side with Bcl6, overexpression of Bcl6 overcomes deficiency of TCF-1, indicating that TCF-1 acts upstream of Bcl6 in promoting *Bcl6* expression.^76^ Interestingly, ablation of TCF-1 during the late phase of Tfh differentiation is redundant for Tfh ontogeny but is necessary for Tfh-dependent GC B-cell development, which highlights the role of TCF-1 in Tfh cell function in GCs.^76^ LEF-1 recognizes a similar DNA consensus motif to TCF-1. This enables LEF-1 to exploit the same mechanisms as TCF-1 to initiate Tfh differentiation. It has been shown that ectopic expression of LEF-1 enhances the transcription of Tfh regulatory genes *(Il6rα*, *Il6st*, *Bcl6*, and *Cxcr5)* in Th1 cells. In addition, LEF-1-deficient, antigen-specific CD4^+^ T cells fail to initiate Tfh differentiation early during LCMV infection.^75^ In keeping with the similar mechanisms enacted by these HMG factors, LEF-1 and TCF-1 show functional redundancy. Indeed, the defects in Tfh differentiation in *Lef1* and *Tcf7* double*-*deficient mice is more profound than those in single-deficient mice, indicating that LEF-1 and TCF-1 coordinate to regulate Tfh differentiation.^75^ Collectively, these results imply that TCF-1 and LEF-1 act together as key players in tipping the Tfh/Th1 equilibrium and an essential prerequisite for Tfh fate commitment.

### Id transcriptional regulators

Id2 and Id3 are inhibitors of DNA binding proteins and are differentially expressed in Th1 and Tfh cells.^81–83^ The Id family of proteins consists of four proteins, Id1, Id2, Id3, and Id4. They are functional inhibitors that act to reduce the DNA binding activity of E-protein transcription factors (E2A, E2-2, and HEB).^84^ Among the Id proteins, Id2 and Id3 play a profound role in the ontogeny of several immune cells, including innate lymphoid cells, regulatory T cells, natural killer cells, invariant NKT cells, and effector and memory CD8^+^ T cells.^85^ In addition, IL-12 signaling negatively regulates Id3 expression in antigen-specific CD8^+^ T cells. However, IL-2 signaling via STAT4 and STAT5 positively mediates Id2 expression. These STATs were found to be enriched at the Id2 promoter in CD8^+^ T cells.^81^ In comparison, little is directly known about the environmental cues that influence Id2 and Id3 expression in CD4^+^ T cells. Although it is likely that Id proteins in CD4^+^ T cells require similar cytokine regulatory pathways as those in CD8^+^ T cells, further studies are needed to investigate this hypothesis. Naive CD4^+^ T cells express high amounts of Id3. During LCMV infection, Tfh cells retained high levels of Id3. In contrast, Th1 cells preferentially expressed Id2.^83,86^ Impaired Id2 expression in viral-specific CD4^+^ T cells restricts T-bet expression and the expression of other Th1-associated genes (granzyme B and IFN-γ), which results in a reduced Th1 population.^86^ Moreover, in the absence of Id2, E proteins circumvent Id2-imposed inhibition and induce several key Tfh genes, including *Cxcr5*, *Il6ra*, *Tcf7*, and *Lef1*, resulting in Th1 cells adopting a strong Tfh signature gene profile.^86^ Nevertheless, it is unclear how Id2 regulates T-bet expression. In this model, it is plausible that high TCF-1 and LEF-1 expression feeds back to downregulate T-bet expression, as mentioned earlier. TCF-1 and LEF-1 play a key role in curtailing T-bet expression. Indeed, Bcl6 expression is unchanged in Id2-deficient Th1 cells, suggesting that Id2 regulation of the Tfh/Th1 differentiation axis may occur exclusively via Tcf7/Lef1 mechanisms and not through the direct regulation of either Bcl6 or T-bet.^86^ Furthermore, Id3 negatively regulates Ascl2-mediated CXCR5 expression. Ascl2 acts by upregulating Cxcr5 and downregulating canonical Th1 gene (*Il12rb1, Tbx21, Ifnγ*, and *Gzmb)* transcription by directly binding to their loci.^87^ Therefore, Id3 deficiency in CD4^+^ T cells promotes Tfh development, which has been observed both in viral infection^86^ and following immunization.^87^

### STAT family transcription factors

The transcriptional regulators of the Janus kinase/signal transducer and activator of transcription (JAK/STAT) family are central to the Tfh and Th1 bifurcation. This pathway is initiated when cytokine ligands bind their respective cognate receptors; as a result, a conformational change occurs that induces receptor rearrangements, leading to JAK activation. JAKs phosphorylate STATs, which then translocate to the nucleus, where they either silence or activate several transcriptional programs.^88^ Therefore, JAK/STAT signaling is an important mechanism by which cells integrate external environmental signals. Interestingly, STAT4 appears unbiased toward differentiation of either Tfh or Th1 cells during early T-cell differentiation; however, it is required for cells to move past the common precursor stage during differentiation, leading to the promotion of Th1 differentiation.^4,69^ In addition, STAT4 induces IL-21 and IFN-γ expression in CD4^+^ T cells both in vivo and in vitro.^4,69^ Among the STAT family of transcription factors, STAT3 influences the Bcl6–T-bet axis toward Tfh development.^20,89^ STAT3-deficient CD4^+^ T cells fail to differentiate into early Tfh cells.^80^ Furthermore, another study demonstrated a profound defect in Tfh subsets due to loss of STAT3 much later in LCMV infection.^90^ STAT3 positively regulates Bcl6 expression first by directly binding to its promoter. Furthermore, the related zinc-finger transcription factors Ikaros and Aiolos positively correlate with Bcl6 expression in Tfh cells during Th1 polarization and in response to *Listeria monocytogenes* infection.^91^ Mechanistically, these factors act together with STAT3 to form a transcriptional complex at the *Bcl6* promoter that initiates conformational changes in chromatin structure and results in gene activation.^91^ In addition, IL-6 signaling also activates STAT1 in CD4^+^ T cells. STAT1 is additionally activated downstream of type I IFNs (such as IFNα and IFNβ), which enables STAT1 binding in the *Bcl6* locus to contribute to Tfh development in in vitro studies.^92^ The combined deficiency of STAT1 and STAT3 in CD4^+^ T cells leads to a complete failure of early Tfh cell development following viral infection.^80^ STAT5 and STAT3 have common binding sites in the *Bcl6* locus, and the ratio of STAT3 and STAT5 is therefore critical, as in higher STAT5 conditions, similar to the scenario with high IL-2, STAT5 can mask the binding site in the *Bcl6* locus, preventing STAT3-dependent *Bcl6* transcription.^63,90^ Furthermore, STAT5 also upregulates Blimp-1 to indirectly suppress Bcl6 expression during the T-cell priming phase in LCMV infection.^93^ STAT3 can also counter this suppression via its own downregulation of T-bet and CD25, the high-affinity receptor for IL-2, indicating that this pathway not only promotes Tfh differentiation but also deters Th1 differentiation.^80^ Taken together, these findings highlight the complex integration of the cytokine milieu that is mediated by the STAT family. However, some work remains to reveal how this is mediated in humans. Unlike in mice, patients with inborn errors of immunity show a conserved role for STAT3, and STAT3 deficiency leads to reduced Tfh cell numbers; in contrast, STAT1 deficiency does not impair human Tfh differentiation.^94^

### KLF2

Another transcription factor, KLF2, takes a wide range of approaches to impair Tfh and promote Th1 differentiation. KLF2 is immediately downregulated after T-cell receptor stimulation in activated CD4^+^ T cells.^95^ KLF2 expression is maintained at low levels in Tfh cells in contrast to non-Tfh cells in viral infection and after immunization.^95,96^ KLF2 directly binds to the promoter region of *Prdm1*, which in turn increases Blimp-1 expression, which, as described, leads to the repression of *Bcl6* expression to further restrict Tfh differentiation.^96^ In addition, KLF2 is enriched at the regulatory region of the *Tbx21* locus, and overexpression of KLF2 results in an increased number of T-bet^+^ and fewer Bcl6^+^ antigen-specific CD4^+^ T cells following immunization.^96^ In addition to these direct transcriptional mechanisms, KLF2 also initiates the expression of sphingosine-1-phosphate receptor 1 (S1PR1) and L-selectin (CD62L) in T cells. Expression of these cell surface receptors facilitates T-cell egress and entry into lymphoid tissues.^97^ Low levels of KLF2 in Tfh cells coincided with reduced S1PR1 expression, similar to that seen in memory CD8^+^ T cells, which downregulated S1PR1 to establish a tissue-resident pool.^96,98^ This suggests that downregulation of KLF2 in Tfh cells may be essential to block egress from lymphoid tissues and to encourage localization within the GCs. In addition, the transcription factor FOXO-1 further acts to promote *Klf2* transcription by binding to its promoter in human T cells, leading to Th1 differentiation.^99^ Conversely, T- and B-cell crosstalk facilitated by ICOS-ICOS-L interactions allows the development of early Tfh cells to overcome KLF2-mediated T-cell migration through the inhibition of FOXO-1.^39,95^ Furthermore, KLF2 has recently been confirmed to be part of the Bcl6-repressed transcriptional circuitry, along with TCF-7, which inhibits key Tfh genes encoding PD-1, ICOS, CD200, IL-6Rα, IL-21, and IL-4.^25^

### Control of Th1 and Tfh identity and plasticity via interacting gene networks

Collectively, a secondary set of transcription factors help coordinate Bcl6 or T-bet expression in Tfh/Th1 cell precursors. These factors act both upstream and downstream of the central lineage-defining factors to tune their expression (Fig. 2). As a consequence, the ratio between Bcl6 and T-bet is disturbed, and precursor cells adapt a fate determined by the dominant lineage-specifying transcription factor. While some connections between different transcription factor families have been established, it is important to understand how these transcription factor networks act together to guide the cell toward one differentiation fate or the other. Furthermore, in accordance with the hypothesis that the balance of T-bet and Bcl6 determines cell fate, the loss of T-bet in T cells promotes Tfh ontogeny at the expense of Th1 differentiation both in vitro and in multiple Th1-biased infection models (including *Toxoplasma gondii*, *Plasmodium berghei* ANKA, and influenza).^4,46,100^ However, it remains important to investigate how the cumulative effect of these secondary transcription factors impacts gene signatures and functional consequences within Th1 and Tfh cells. The relationship between these transcriptional regulators during in vivo T-cell differentiation is starting to emerge,^25^ and it is likely that these transcription factors may play distinct roles within each effector subset. Similar to their behavior in a Th1-biased environment, in a Th2- or Th17-biased cytokine milieu, Tfh cells differentiate in parallel with Th2 and Th17 cells. Tfh cells can secrete multiple cytokines, such as IL-21, IL-4, IL-2, IL-9, IL-10, IL-13, and IFN-γ. It is now apparent that distinct functional Tfh subpopulations hold the potential to produce specific combinations of cytokines.^2,9,96,101–104^ Indeed, Tfh subpopulations have been described both in humans and mice, and these may play distinct roles in mediating humoral responses in allergy and asthma and lead to specific protection following vaccination and infection.^94,102,105,106^ In a key study, Eisenbarth and colleagues demonstrated that IL-13-producing Tfh (Tfh13) cells co-express Bcl6 and GATA3 in mice and humans with IgE antibodies against allergens. Tfh13 cells triggered the production of high-affinity IgE, which led to the induction of anaphylaxis.^102^ In addition, over the course of Th2-skewed infection (with the helminth *Nippostrongylus brasiliensis*), the cytokine profile of Tfh cells changes from one dominated by the production of IL-21 to one favoring IL-4-producing Tfh cells. These subsets are transcriptionally, phenotypically, and functionally distinct and provide different helper signals to GC B cells, suggesting that the role of Tfh cells may change dynamically over the course of an infectious challenge.^107^ However, we are only starting to appreciate this functional heterogeneity and plasticity within Tfh populations and potentially other T effector lineages. Furthermore, within mature Tfh and Th1 populations, it is unclear how epigenetic regulators establish and maintain the balance of lineage-defining transcription factors. As the *Bcl6* and *Tbx21* loci are kept in a permissive histone state within mature Tfh and Th1 cell pools, respectively, it is apparent that these gene networks remain responsive to changing environmental cues throughout infection.^10,108^ This likely acts to maintain identity and function or to generate plasticity between CD4^+^ effector populations and may mediate the functional heterogeneity within Tfh and Th1 populations throughout infection and disease.^102,105,107^ Thus, the integration of and balance between transcription factor instruction with inflammatory mediator signaling is of undeniable interest.

## Cytokine mediators of the Th1/Tfh dichotomy: translating inflammation into cell fate

**Fig. 3:**
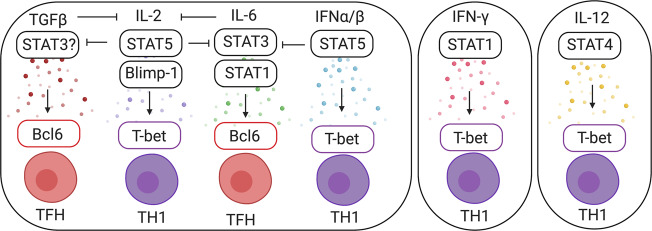
Environmental cues that instruct CD4+ T cell differentiation. Individual cytokines activate downstream transcriptional factors, which in turn regulate Bcl6 or T-bet expression to imprint either the Tfh or Th1 fate

We recently reported the transcriptional heterogeneity that underlies the flexibility in Tfh differentiation in distinct viral infections.^46^ Specifically, T-bet is required in a context-dependent manner for Tfh cell generation during LCMV and influenza infection. The essential role of T-bet is determined by variations in its expression level that are set by distinct inflammatory cues in these individual infection types.^46^ The impact of the distinct cytokine milieu in various viral infections was confirmed in a study that investigated the inflammatory cytokines that distinctly impact Tfh and Th1 differentiation during vesicular stomatitis virus and LCMV infection.^109^ Taken together, these works propose that each viral infection elicits unique inflammatory cues that work independently or together to regulate transcriptional networks that promote either T-bet or Bcl6 and control the Th1/Tfh bifurcation (Fig. 3).

### IL-6

IL-6 was one of the first indispensable cytokine signals shown to drive Tfh cell formation.^80^ This multifunctional cytokine is secreted by various cell types, including DCs, T and B cells, macrophages, fibroblasts, endothelial cells, glial cells and keratinocytes.^110^ In LCMV infection, IL-6 deficiency impaired early Bcl6 expression and Tfh differentiation.^80^ IL-6 signals through STAT3, which is sufficient to trigger initial Bcl6 expression and the Tfh fate trajectory. However, STAT3 deficiency does not directly phenocopy the outcome of IL-6 deficiency in Tfh development, implying that an alternate signaling cascade is needed to maintain Tfh fate commitment during LCMV infection. In addition to STAT3, IL-6 stimulation also activates STAT1 in CD4^+^ T cells.^111^ Antigen-specific CD4^+^ T cells dually deficient for STAT1 and STAT3 completely fail to form Tfh cells, replicating the outcome of IL-6 deficiency and confirming that STAT1 and STAT3 act in collaboration downstream of IL-6 signaling to promote Tfh ontogeny.^80^ Of note, during infection, cytokines such as IL-6 are not present in isolation. IL-6 has additional signaling mechanisms to guard against other factors that may promote Th1 differentiation. Indeed, via STAT3, IL-6 also negatively regulates the surface expression of CD25 during LCMV infection.^80^ As mentioned earlier, high IL-2 sensitivity inversely correlates with Bcl6 expression and Tfh differentiation;^112^ this dual signaling is therefore important to reinforce IL-6-directed Tfh differentiation. IL-6 is secreted by conventional DCs in response to CD40 stimulation and in the presence of type 1 IFN in viral infection.^113^ In a recent study, the timing of IL-6 production by DCs was shown to direct the Tfh/Th1 dichotomy, whereby an early wave of type I IFN induced DCs to produce IL-6. In turn, this promoted precursor cells to commit to the Tfh fate at the expense of the Th1 fate.^109^ In contrast, late production of type I IFN resulted in DCs becoming insensitive to type I IFN; hence, DCs failed to produce IL-6. In this setting, precursor cells adopted a Th1 fate trajectory instead of a Tfh trajectory.^109^ Another study showed that activated follicular B cells also secrete IL-6 early in influenza infection, which is sufficient to drive Tfh differentiation.^114^ In accordance with these findings, within GCs, follicular DCs produce IL-6 in the late stage of Tfh development.^115,116^ Critically, this late induction of IL-6 is required and sufficient to clear chronic infection.^117^ Interestingly, previous studies have reported that in the absence of IL-6, there is no difference in the frequency of Tfh cells between early LCMV infection and later points after the acute phase.^118,119^ Taken together, these studies confirm that while IL-6 does promote Tfh differentiation, this is regulated in a context-dependent and spatiotemporal manner between and during infection.

### IL-21

IL-21 is the cardinal Tfh cell-derived cytokine. While IL-21 alone is not required in regulating Tfh differentiation, in combination with IL-6, IL-21 promotes Tfh differentiation by activating the STAT3 signaling cascade in vitro.^20,119^ IL-21 is a part of the IL-2 family of cytokines, including IL-2, IL-4, IL-7, IL-9, and IL-15, that share the common γ-chain (*γ*_*c*_) IL-2R subunit.^120^ Both IL-6 and IL-12 can induce IL-21 expression in murine CD4^+^ T cells in vitro and in vivo.^4,121,122^ However, mice deficient in IL-21R have no defect in Bcl6 expression or in Tfh differentiation during viral infections.^80,114,123^

### TGF-β

Transforming growth factor-β (TGF-β) augments Bcl6 and deters T-bet expression. This tips the Bcl6–T-bet equilibrium in favor of Tfh differentiation at the expense of Th1 differentiation.^124^ TGF-β receptor-deficient CD4^+^ T cells had higher levels of IL-2Rα expression and STAT5 activity than wild-type cells in the early stage in LCMV-infected mice,^125^ indicating that TGF-β restricts IL-2 responsiveness before CD4^+^ T cells bifurcate into Tfh or Th1 cells. As a result, there were fewer Tfh and more Th1 cells in the absence of the TGF-β receptor after viral infection.^125^ Human naive T cells cultured in the presence of TGF-β demonstrated increased expression of Tfh-related genes, including BCL6 and CXCR5, and decreased expression of Blimp-1.^124^ TGF-β signals through STAT3 to drive the differentiation of human Tfh cells;^124^ however, this has not been established in mice. In keeping with this essential role, TGF-β signaling in Tfh cells is critically required for GC formation and for the generation of influenza-specific antibodies.^125^

### IL-2

IL-2 is a well-documented inhibitor of Tfh differentiation.^93,126^ Currently, the expression of CD25 (the α chain of the high-affinity IL-2 receptor) is the earliest marker of cell fate between Tfh and Th1 cells. Early in infection, CD25 expression is downregulated in early Tfh precursors, making the cells insensitive to the inhibitory effect of IL-2 signaling. In contrast, the opposite is true for early Th1 precursors, which can be distinguished by their expression of CD25.^7^ Thus, cells with higher CD25 expression are destined to become Blimp1^+^T-bet^hi^IFN-γ^hi^ Th1 cells,^7^ while cells with lower CD25 expression are early Tfh precursors, which are fated to become Bcl6^hi^CXCR5^hi^ Tfh cells.^7^ Recently, DiToro et al. showed that the expression of IL-2 coincided with that of Bcl6 in CD4^+^ T cells.^6^ Thus, this established a new paradigm whereby early Tfh precursors are IL-2 producers and the Th1 precursors that express CD25 early are the IL-2 consumers.^6^ The equilibrium between IL-2 producers/consumers maintains IL-2 levels, and any imbalance leads to either the promotion or inhibition of Tfh differentiation. We recently reported that T-bet-deficient CD4^+^ T cells adopt the Tfh fate instead of the Th1 fate in influenza infection.^46^ However, the role of T-bet in Tfh cells is not recapitulated during LCMV infection.^46,69^ We proposed that there is a difference in IL-2 and subsequent STAT5 activity between these settings. Indeed, CD25 expression and STAT5 activity are augmented in LCMV infection in comparison with influenza infection.^46^ In the absence of T-bet during LCMV infection, the loss of IL-2 consumers (Th1 cells) results in excessive IL-2 and STAT5 activity, and thus, cells are blocked from both the Th1 and Tfh fates.^46^ Together, these results explain how high IL-2 may regulate Bcl6 expression to act as a cytokine switch between Tfh and Th1 fate commitment. In contrast, during influenza infection, there is likely a competing cytokine, such as IL-6, that may allow the Tfh fate to be promoted, even when IL-2 consumers are lacking in the absence of T-bet.^46^ Consistent with this, recent research led by the Ballesteros group found that GC Tfh cells utilized cell-intrinsic IL-6 signaling, which blocked STAT5 from binding to the *II2rb* locus (encoding CD122, a chain of the IL-2 receptor); in turn, GC Tfh cells lacked expression of the IL-2 receptor and remained insensitive to IL-2.^127^

### Type I IFNs

Type I IFNs (IFN-α and IFN-β) regulate the STAT5 signaling pathway in CD4^+^ T cells to promote Th1 differentiation while inhibiting the Tfh fate trajectory.^90^ Type I IFNs are a pleiotropic family of cytokines that regulate cell-type-specific signaling pathways. These cytokines signal through the type I IFN receptor, which consists of two subunits, IFNAR1 and IFNAR2.^128^ IFNAR-deficient cells have increased Bcl6 expression and a Th1-like gene profile,^90^ and CD4^+^ T cells treated with type I IFNs show increased CD25 expression and STAT5 activity. STAT5 activity is a robust inhibitor of Bcl6 expression and promotes Blimp-1 expression in CD4^+^ T cells.^63^ By blocking type I IFN signaling, the Tfh phenotype is partially recovered in STAT3-deficient CD4^+^ T cells during LCMV infection. This indicates that the STAT3 and type I IFN signaling pathways have an opposite role in Tfh development following viral infection. Concurrently, STAT3-deficient Tfh cells have elevated expression of a number of IFN-stimulated genes.^90^ However, as discussed above, the immune population directly responding to type I IFN is critical, as signaling through IFNAR1 on DCs leads to enhanced IL-6 production, which subsequently promotes the Tfh fate.^109^

### IL-12

It has been known for more than two decades that the IL-12-STAT4 signaling cascade promotes T-bet expression, which directs Th1 development.^129^ The idea of IL-12 initiating both Tfh and Th1 differentiation was first observed in human CD4^+^ T cells.^130,131^ Later, murine CD4^+^ T cells cultured in IL-12 demonstrated the expression of both canonical Th1 (IFN-γ and T-bet) and Tfh markers (IL-21 and Bcl6).^4^ This suggests that both Tfh and Th1 cells have a common transitional state of differentiation. Although IL-12 predominantly acts through STAT4 signaling, STAT4-deficient CD4^+^ T cells exhibit impaired Th1 but intact Tfh differentiation, potentially suggesting a temporal role for IL-12 in Tfh differentiation, where it is not needed late during infection.^4,69^ In addition, IL-12-dependent STAT4 signaling is required for the expression of T-bet, IL-21, and IFN-γ in Tfh cells, indicating a role in fine-tuning Tfh subpopulations.^69^ In addition, T-bet expression can also be induced by IFN-γ-STAT1 signaling in an IL-12-independent manner both in vitro and in vivo.^4,132,133^ Interestingly, however, despite the close association between IFNγ, IL-12, and Th1 differentiation, dual deficiency of Ifnγr1 (the IFN-γ receptor) and STAT4 still permitted the expression of T-bet during *Toxoplasma gondii* (*T. gondii*) infection. Another IL-12-related cytokine, IL-27, binds to WSX-1, a class I cytokine receptor family member that shares similarities to the IL-12 receptor, and this signaling pathway has been shown to play a role in STAT1-dependent T-bet induction.^134^ Taken together, these findings underscore that the unique cytokine milieu is critical in the regulation of lineage-defining transcriptional factors and, in turn, the tailoring of the Tfh/Th1 bifurcation in pathogen-specific ways.

## Concluding remarks

At present, it is not clear whether dominant inflammatory cytokines create an overwhelming milieu in which lymphoid organs are awash with a key, specific cytokine. More likely, however, niches exist within organs in which cytokines are restricted due to their precise expression by distinct cellular sources.^109,135–137^ The DC sources of these cytokines that act to facilitate Tfh and Th1 fate commitment have been recently reviewed elsewhere.^127,136^ It is, however, important to note that the cellular sources of cytokines, such as IL-12 and IL-6, are not restricted to a single DC subset. Therefore, continual changes in cytokine stimulation and fate directing through multiple T cell–DC interactions may regulate the gene regulatory programs that ultimately tip the balance between Tfh and Th1 differentiation.^138^ Furthermore, it remains unclear how individual cell interactions or cytokines regulate heterogeneity within Tfh subpopulations, and how this is dynamically regulated in the leadup to or within GC reactions.^2,102,107^

As described herein, the balance of the type I IFNs IL-6 and IL-2 appears to be essential to govern the transcriptional networks that promote either Bcl6 or T-bet and shape the Tfh and Th1 dichotomy. Ultimately, these are the key cytokines that establish the equilibrium between the promotion of humoral or cellular adaptive immunity.^46,109^ While several studies have linked the context-specific interplay between cytokine environment and CD4^+^ T-cell differentiation, there is still a gap in knowledge regarding how these cytokines are differentially regulated in a pathogen-specific manner. Of note, these considerations appear to be of critical importance for understanding the immunopathogenesis in the current Coronavirus Disease 2019 pandemic. Profiling T cell responses of severe COVID-19 patients has demonstrated elevated T-bet expression which coincides with limited TFH differentiation.^139,140^ A study conducted in 2003 that treated patients with type I IFNs during active severe acute respiratory syndrome coronavirus 1 (SARS-CoV-1) infection showed promising results for the promotion of viral clearance.^141^ Furthermore, a trial blocking IL-6 to disrupt the cytokine storm in severely affected SARS-CoV-2 patients is underway.^142^ While our current knowledge of the interplay between cytokine and transcriptional gene networks would suggest that both of these immunotherapies heighten Th1 differentiation, this may come at the cost of Tfh differentiation and therefore be detrimental to the development of neutralizing humoral immunity.^2^ Thus, understanding how altering the cytokine milieu directly modulates the multiple gene transcriptional networks that underpin CD4^+^ T-cell differentiation in a pathogen-specific manner is of critical importance when considering new therapeutic targets to both promote viral clearance and drive protective immunity.
